# What Price Screening?—II
*This paper comprises the thoughts of the author, strongly influenced by the stated literature, and does not necessarily represent the opinion of the South-Western Regional Hospital Board


**Published:** 1970-04

**Authors:** N. A. Dent

**Affiliations:** Assistant Senior Medical Officer, S.W. Regional Hospital Board Lecturer in Medical Statistics, University of Bristol


### What Price Screening? — II*

N. A. Dent, M.B., Ch.B., D.P.H., D.Obst.R.C.O.G.

Assistant Senior Medical Officer, S.W. Regional Hospital Board

Lecturer in Medical Statistics, University of Bristol

"Thou art weighed in the balances and art found wanting"—Daniel, Chap. 5, Verse 27

Disease is a continuing process and to divide it into phases imposes somewhat arbitrary classifications on it. From conception to the grave an individual is subject to a variety of stimuli which disturb his equilibrium and may well leave permanent signs of their impact. Such signs may be precursors to, and useful signals of, pre-disposition to subsequent disease. All individuals may be regarded as being in post-convalescent phases of previous overt and occult disease and in the pre-symptomatic phases of those yet to come.

In health evaluation, as in so many other fields where few absolute norms exist, detecting changes in status is easier than defining absolute levels. The proximity of the next disease may be heralded by a change in the intensity of some signal. Doctors have always varied in the amount of notes they keep. Relatively good control of a single spell of illness is perhaps more than possible without much recording, but the evolving health status of a patient may be prejudiced by inadequate recording of observations. In making a diagnosis, critical decisions have to be made about the redundancy of information collected, and, in terms of present storage media about the proportion of apparently marginally valuable information that can be kept for any length of time. Indeed, it is impossible to conceive an economic record of all events which might achieve the earlier differential diagnosis of impending illness.

Out of this vast spectrum of scales of morbidity the concept of need for medical evaluation and care emerges. When made, the medical diagnosis can be regarded as a convenient synopsis of a set or sub-set of many facts. The total amount of need for care can be regarded as being broadly determined by the medical authorities. On the other hand, the demand for care by patients will be influenced by the interaction of illness with other factors and an individual may demand more attention than he really needs (Feldstein 1966). Conversely, less care than is really needed may be requested because a potential patient is unaware of the value or even existence of appropriate services.

Once a patient is "found" by the health services, his consumption of specific items of investigation and care will largely be determined by professional "brokers". The treatment a patient will receive will depend upon his professional adviser's knowledge of the availability of, and possible interchange of, alternative types of care. A result of adoption of screening procedures is to extend the philosophy that a doctor knows best for the patient to a stage even before the latter is at present prepared to accept that anything is wrong or a need exists.

That the community's definition of need is not necessarily that of its professional advisers is demonstrated in the proliferation of patent medicines, osteopaths, chiropractors and faith healers. Not only is the community view of health and need for care different from that of the profession but the norms for both are to some degree a matter of social definition and change from place to place and time to time. In the eyes of the village maidens the charms of gilded dukes and belted earls were surpassed by those of W. S. Gilbert's Pale Young Curate. Recently there has been a tendency for criminals to receive treatment rather than punishment, though we still remain some distance from the situation envisaged in Samuel Butler's "Erewhon" where doctors treat crime and policemen illness.

It is likely that no standard set today will be fully acceptable ten years hence. All that we may confidently anticipate is that the total expectancy for care will always exceed our capacity to meet it, and that doctors will continue to find individual patient care and community expectation of available care often confounded. Patients with coronary thrombosis and patients with cerebral thrombosis may have an equal need for a hospital bed. The fact that the former have greater prospects of early recovery may make their admission more compatible with the community wish that hospitals will continue to have beds available to accept emergencies.

Three decades ago such conditions as poor sight or deafness or toothlessness were regarded as normal facets of ageing. Ageing is inevitable in biological systems as in machines: indeed it has been defined as that adverse state of the organism which no input can remedy (Boulding 1965). Although certain accelerators of the ageing process are known, so far nothing capable of postponing it is recognized. Because it is instrumental and has no value in itself, a point can be estimated, in the absence of technological change, at

*This paper comprises the thoughts of the author, strongly influenced by the stated literature, and does not necessarily represent the opinion of the South-Western Regional Hospital Board.

which a machine should be replaced. In human beings however the problems of ageing present delicate issues in the field of need for medical care.

In attempting to estimate the extra charge on health services which screening techniques might impose it is necessary to enumerate the morbidity at present known to the hospital and other services—by no means a simple task. Exactly a hundred years ago in 1870 Florence Nightingale reported to the Fourth International Statistical Congress:—

> "Every hospital has followed its own nomenclature and classification of diseases, and there has been no reduction on any uniform model of a vast amount of observations which have been made in these establishments. So far as relates either to medical or sanitary science, these observations in their present state bear exactly the same relation as an indefinite number of astronomical observations made without concert and reduced to no common standard, would bear to the progress of astronomy. The material exists, but it is inaccessible."

Since that time the international classification of diseases has evolved to add some extra precision to statistical knowledge of disease, and data is regularly and routinely published under diagnostic headings, as for example, in the Registrar General's Annual Statistical reviews and the Hospital Inpatient Enquiry. However, these publications still suffer in that they enumerate morbidity only approximately. Clearly not all diseases are regularly fatal or cause hospital admission. Similarly the absence of provision for multiple cause coding allows only one disease of a cluster to score in mortality statistics. Alderson and Dent in 1966, using evidence assembled in the inter-American mortality investigation in which Bristol participated, found that in almost half of 99 diabetics dying during the study period, no evidence of diabetes was recorded on the death certificate.

A similar deficiency occurs in Hospital In-patient Enquiry data, which, plus the fact that duplication occurs, renders these figures poor measures of disease prevalence. New out-patients amount to 50% more than total in-patient spells but for them no diagnostic registers exist. Nor do laboratory or other hospital service departments regularly keep such lists.

There remain a large number of patients treated by their General Practitioners who will be largely unknown to local hospital services. Migration of patients could lead, say, to the inward transfer of a number of diabetic patients who have been investigated elsewhere, and whom the General Practitioners are able to continue to treat more than adequately without reference to hospitals.

It follows that the precise number of patients with lesions possibly amenable to screening who are at present attending hospital or General Practitioner services is largely unknown. Even if the difference between patients now known and those who could be discovered by screening could be estimated it would probably be an error to assume that the "new cases" found would require similar investigations, surveillance and treatment to that received by those already known. It seems reasonable to assume, for instance, that all diabetics really needing hospital in-patient care already receive it and that the pattern of care of the extra (marginal) cases will be different. Similarly cases of glaucoma found by screening will require different surveillance patterns from those who present, as acute cases, now. Even in what seemed to be a fairly indisputable field, the early diagnosis of carcinoma of the cervix, some experts now suggest that the advanced malignant state is by no means an inevitable sequel to previously accepted appearances of carcinoma-in-situ, and we may expect some variation in follow-up routine.

A patient who suddenly surrenders to medical services for one condition is theoretically available to be screened for precurses of others. Like other services the hospital, in theory, contains ample opportunities for screening. A basal cell carcinoma might be discovered, in the course of routine examination, in a patient admitted for an operation on his inguinal hernia. However, whether a retinopathy would be discovered is doubtful. The dilemma of redundancy of collected information is apparent again. The somewhat arbitrary order of examination might only lead to retinoscopy after firstly glycosuria and then a raised blood sugar had been found.

Again it can be argued that specimens collected for one purpose might be examined in depth. For instance, specimens received by the Blood Transfusion Service offer an opportunity of gathering information about the incidence of communicable and non-communicable disease present or predictable, e.g. latent liver disease, venereal disease, salmonellosis, brucellosis, tumours, cardiovascular, endocrine and metabolic disorders, in addition to those of the blood itself.

Whether the early translation of physiological need to medical care will result in higher or lower total demand is difficult to foretell (Feldstein 1966). A person who anticipates the onset of ill-health may create relatively large expenditures for preventive services and only postpone expenditure on treatment. Early operation at a curable stage may well result in excess total demand by the patient for services in connection with later and possibly unrelated conditions.

In infectious diseases such as venereal disease and tuberculosis it is certainly desirable to identify patients before they exhibit symptoms so that treatment may be effected and measures taken to prevent spread. With non-communicable diseases, those who are not examined receive no direct benefit. Nor can there be indirect benefits to individual health or to the National Economy other than those stemming from increasing epidemiological knowledge of the early stages of the disease.

The recent growth of medical literature often seems to imply that no price is too great to pay for good health. However, many are unwilling to pay even a small price. Many do not brush their teeth even when they recognise that to do so might significantly reduce caries. Though ignorance may explain the failure of some to take appropriate steps, this is clearly not the reason in many instances for there are doctors who continue to smoke: and physicians who are no more likely to come forward early in malignant disease than lay persons (Blackwell 1963, Sutherland 1960).

Just as they differ in the relative values they place on other commodities, people, apparently, differ in the value they place on health. To suggest that all should use the same amount of a facet of health services seems likely to result in some misallocation of resources (Fuchs 1966). All attendances at medical centres may be regarded as inconvenient to the patient. When he is ill, medical consultation is a priority and the value of his time which might be used in other ways is low. However, in estimating the cost of periodical medical examinations and routines it would be an error to ignore the cost of inconvenience to the patient (Becker 1965). Another area in which costs may rise is that of illness which is at present incidental. To extend medicine into the presymptomatic stages of illness is hardly consistent with appeals to the public not to trouble doctors with minor complaints. The doctor who is seen to have time to conduct screening may seem to have little to do!

In stretching out into the fields of possibly redundant information the law of diminishing returns must apply, unless the screening tests themselves become more and more conclusive. However, experience in some areas suggest that rather complicated surveillance or investigation may be required after the initial selection by screening, as for example the need for "special investigation centres" in developmental paediatrics. Again, as any subject becomes larger, more and more specialization is created on its margins. However, to change people is probably much more difficult than to change their environment and it is obvious that for screening and its follow-up to be widely accepted, inconvenience must be minimised and, for example, convenient hours and distances to receive the service are paramount. In more senses than one, staff may well be more mobile than many of the patients qualifying for continued surveillance.

We should accept the onus of proof that we can guarantee some kind of help before advocating screening. Furthermore, whenever considering greater expenditure on health services we ought to be prepared to show that the return is likely to be greater than that which might accrue from other forms of investment (Fuchs 1966, Cochrane 1967). Although on different time scales, expectant mothers and the ageing may each gain from frequent surveillance and by preventive actions by doctors but they may also benefit variously from better housing, from better diet, from not having to go out to work, from having someone to help with housework or simply from company.

The soldier in "The Tinder Box" soon realized what a capital tinder-box it was. "If he struck it once the dog came who sat upon the chest of copper money; if he struck it twice the dog came who had the silver; and if he struck it three times there appeared the dog who had the gold." Dr. Rowland urges us to regard our early screening enterprises as research ventures and approach them scientifically. Before embarking upon wider screening services let us be sure we recognize the gold and have to hand a tinder box whose properties are proved.

For centuries people have consulted doctors about their ills, and this despite the fact that many of the prescribed remedies have subsequently proved to have been irrelevant. Doctors may have received credit in the past where often it possibly belonged to nature. That the profession influences and is influenced by community expectation of health, and conversely health services, has been discussed. The population must not now set itself such low thresholds for disease that impossible demands are made for services, whilst, almost by definition, it "feels less and less well enough" to work and thus be sufficiently productive to finance the service (Teeling-Smith 1969).

### REFERENCES

**Alderson, M., and Dent, N. A.** (1966) unpublished.

**Becker, G. S.** (1965). A theory of the Allocation of time—The Economic Journal **75,** 493.

**Blackwell, Barbara** (1963). The Literature of delay in seeking Medical Care for Chronic Illness—Health Education Monographs **16,** 3.

**Boulding, K. E.** (1965). The Menace of Methusalah — Journal of Washington Acad. Sci. **55,** 171.

**Cochrane, A. L.** (1967). Publ. Hlth. (Lond.) **81,** 207.

**Feldstein, P. J.** (1966). Research on the demand for Health Services—Millbank mem. Fd: Quart. **44,** 128.

**Fuchs, V. R.** (1966). Contribution of Health Services to the American Economy—Millbank mem. Fd. Quart. **44,** 65.

Nightingale, Florence 1870 quoted in The Health of the Community by **C. Fraser Brockington** (1965) (J. A. Churchill) p. 296.

**Sutherland, R.** (1960). Cancer: the significance of delay (Butterworths) p. 196.

**Teeling-Smith, G.** (1969). Brit. Hosp. Journal & Soc. Serv. Review. 1699.

